# Changes in Communicating Bad News in the Context of COVID-19: Adaptations to the SPIKES Protocol in the Context of Telemedicine

**DOI:** 10.3389/fpsyt.2020.599722

**Published:** 2020-11-23

**Authors:** Jucier Gonçalves Júnior, Thalya Gonçalves Lôbo do Nascimento, Myllena Maria de Morais Pereira, Elysyana Barros Moreira

**Affiliations:** ^1^Department of Internal Medicine, Santa Casa de Misericórdia, Fortaleza, Brazil; ^2^School of Medicine, Universidade Federal Do Cariri, Barbalha, Brazil

**Keywords:** coronavirus infection, health communication, death, mental health, palliative care, public health, SPIKES protocol

## Introduction

The Death/Dying process (DDP) has profound repercussions on the mental health of patients, family members and friends submitted to it. These consequences bring fear, sadness, feeling of loneliness, abandonment and anguish (1). The representations of this process depend on social, economic, political and historical factors in which that community is inserted. Tools such as the humanization of care based on active listening, assertive communication and a good doctor-patient relationship are essential to face the difficulties inherent in terminality (2). However, several studies have already demonstrated difficulties on the part of health professionals in dealing with issues related to terminality. Therefore, these professionals have difficulty in the realization of effective communication (3).

Since the start of Pandemic by the COVID-19, to date, more than 761,779 deaths have occurred and more than 20 million people have been infected (4). Due to the high infectivity of the virus, patients are separated from family and friends (5). Professionals are forced to transmit news, including bad news, impersonally and without prior training via mobile telephony. Thus, conversations using online communication systems have an increasing role in palliative care. These online telecommunications-based services challenge effective communication and communicating bad news (CBN). Tools such as the SPIKES protocol (6) are essential to operationalize care and prepare professionals for a more welcoming, effective and less impactful approach.

Robert Buckman (1992) created the SPIKES Protocol in order to guide health professionals in communicating bad news to patients and their families. The protocol is divide in steps: (a). This procedure consists of a six-step mnemonic: S - Setting up; P - Perception; I - Invitation; K - Knowledge; E - Emotions; S - Strategy and Summary (6). However, with the advent of the COVID-19 pandemic there was an overload of the health system. The health team's ability to perform welcoming work, active listening and care were harmed.

In this context, strategies and protocols designed to establish professional security in terminal situations, such as the SPIKES Protocol, are difficult to implement. Thus, health professionals worldwide face an unprecedented challenge (7, 8)—how to establish care without touching, without eye contact, without physical presence? How to mourn without seeing the bodies? How do you bond with people you don't know without being there? Therefore, health teams have been adapting and incorporating the use of social media in practice making the implementation of protocols such as SPIKES challenging (3, 6, 9, 10).

Therefore, the aim of this article was to suggest an adaptation of the SPIKES protocol for CBN in the management of patients hospitalized with COVID-19 when the therapeutic interventions are limited. In particular, we will use scientific literature on the COVID-19 related challenges to highlight key points of adaptation to the SPIKES protocol.

## A Proposal for The Adaptation of The Spikes Protocol for Remote Communication in The COVID-19 Pandemic

### (S): Setting Up

Preparing for communication. At this step, the physician thinks about how to speak, the possible reactions of family members and how to deal with them and prepares the place for the conversation (6).

Some health professionals, who previously had no direct contact with DDP, are having to deal with this situation daily and intensely during the pandemic. The DDP in Western culture it is still taboo (3) and a representation goes back to the conception of death adopted from the eighteenth century (5). It is synonymous with failure/fallibility, impotence, sadness, suffering and abandonment (3). Besides that, the uncertainties about the natural history of COVID-19, the lack of scientific information and the experimental protocols cause psychological distress in the team, compromising care and the realization of protocols such as SPIKES. Steps like “S” (Setting Up), “P” (Perception), and “I” (Information), for example, are impaired in the protocol. How will I prepare the environment well if family members are sometimes in a distant neighborhood or even in a different city? Interactions through social media? How to provide information safely if the knowledge produced so far is scarce? (6, 10). So in the step “S” (Setting Up), in the context of the pandemic, we advise that:

- There is a suitable place where the professional can make the calls and/or video calls, if possible even outside the COVID-19 unit so that he can remove the PPE, eat beforehand and go to the bathroom, if necessary. It is advisable to use video calls at CBN due to the possibility of identifying, on the part of the professional, non-verbal signals coming from the receiver in order to direct the conversation (10);
- Prepare a script listing the main topics of the patient's evolution, his name and the name of the companion who usually answers the phone. It is important to emphasize that, in search of success in communication, it is necessary to have a deep knowledge about the clinic and the patient's demand (11, 12);
- Organize, according to the demand, the main priorities for the discussion (those he perceives will be more delicate information should be prioritized) (6, 10).

### (P): Perception

It is necessary to perceive the physical and psychological state, the expectations and understanding of the companion/family member. At this step, the professional tries to realize how much the patient/family understood the disease and corrects misinformation (6). Thus, at this step, perhaps the most challenging for professionals, due to social distance, there is a difficulty in capturing signals and expressions from the recipients of information:

- Call the companion by name, as well as the patient by name. This action helps to consolidate the doctor-patient/family relationship (11–14);
- Always ask if there is someone close to the companion who is receiving the information and advice that, if the companion is unable to keep it going, pass it on (12, 14);
- Notice variations in the companion's tone of voice (tones of doubt, sadness, pauses) (6, 13);
- In the case of a video call, attention to non-verbal language such as crossing arms, frequent deviations in the look. Research has shown that 84.3% of professionals are aware of the role of non-verbal communication and use these cues when they are communicating (13).

### (I): Invitation

Inviting companions to understand the disease. Sometimes, patient's/family members do not want to know details about the disease. At this time, the professional must be available to answer any future questions (6). So, in this step, two elements are fundamental:

- Ask the companions how far they know about the patient's illness, diagnosis, health status and prognosis. This step is analogous to what is seen in face-to-face clinical practice (2, 14–16);
- Make yourself available to answer questions (14, 16).
- Try to create an intimate atmosphere even on the smartphone screen, A safe and welcoming environment, showing willing to help (14).

### (K): Knowledge

Providing information. At this stage it is essential to provide information in a simple way, with accessible language and to be interested in patients' doubts about technical terms.

Conversations using online communication systems have an increasing role in palliative care, as families are faced with so many changes, including reduced face-to-face contact and even abandonment of traditional funeral services. These online telecommunications-based services challenge effective communication and CBN (2, 7, 17). This impersonal process makes it difficult to understand the DDP and the palliative approach because it promotes a reality break—family members deliver a person with shortness of breath to the hospital and find themselves collecting a dead body in a very short period of time (2). In association with that, the potential psychological impact on loved ones and patients can vary from mourning to depression or from feeling of loss to immense guilt for not being physically present at the moment of death (17, 18). In the context of the SPIKES protocol, the steps “E” (Emotions) and “K” (Knowledge) (6) are especially harmed.

Thus, at the “K” (Knowledge) step, it is essential that the health professional mention the medical needs that need to be discussed:

- Offer information about the patient's evolution, diagnosis, prognosis respecting the family's social, economic and cultural limitations (13, 14, 16, 19);
- Avoid using medical jargon (e.g., sedoanalgesia) to the detriment of terms such as “she/he are sleeping under medication” (13, 14, 19);
- Always ask, at the end of each orientation, if the message was heard and understood (2, 6);

### (E): Emotions

Expressing emotions. The professional must offer support and solidarity through a gesture or a phrase of affection. At this step, professional attention is essential:

- One must perceive and become sympathetic to the manifestations of sadness and/or joy, depending on the news, the family members/companions (2);
- Show available and empathetic posture—demonstrate that you understand suffering and difficulties; Given that in many cases after passing on difficult information, people do not actively pay attention to what is said later (12, 14);
- Getting emotional is allowed, it just can't be more than the companions themselves. A survey carried out in 2017 showed that about 40% of doctors feel sad when they had to give bad news (13, 14).

### (S): Strategy and Summary

Before discussing therapeutic plans, it is important to ask the patient if he is ready for this moment. Before discussing therapeutic plans, it is important to ask the patient if he is ready for this moment. It is important to always make it clear that the patient will not be abandoned. And there is a treatment plan thinking about the best that can be offered to him at that moment (6).

Two factors directly affect step “S” (Strategy and Summary). The first is the little time spent on communication. This factor also affects not only step “S” (Strategy and Summary), but also the “S” (Setting Up) and “P” (Perception) (6). This fact rests on two prerogatives: (I) the work overload due to the exhaustive routines of the care units for COVID-19 and (II) the lack of training in palliative care, which is usually reflected in some professional discourse rich in automatic, impersonal and technical information (10). Therefore, the COVID-19 pandemic has led to growing concerns not only about limited medical and hospital resources (e.g.,: ventilators, medications, gauze, and intensive care beds), but concern about the professionals' interpersonal limitations (20).

The second element to be considered is the lack of communication between professionals in the sector. It is not uncommon for the doctor/nurse who gives the news in the daily bulletin to not be the same person who is daily assessing the patient. This lack of consistency causes dissonance in information and suffering to family members due to a feeling of insecurity, because the hospital's restriction on families, *per se*, already contributes to high levels of psychological suffering in the general population and also made it difficult for overworked teams to establish trusting relationships through digital means (21, 22). Thus, the implementation of telemedicine can be a facilitating agent in these meetings with inpatients (19). However, the lack of skills and attitudes about the use of technologies and social media (6, 10) in a stressful environment such as the COVID-19 units can also be a factor that impairs care.

Therefore, at this step, two elements must be taken into account:

- Summarize the information given, preferably repeating it in the same way as it was presented (13–16);
- Announce that the call is coming to an end (3, 6, 14);
- Inform the date of the next call, as well as, if possible, the name of the professional who will be responsible for it. Here, the need to be the same professional is highlighted, in order to maintain continuity of care and relationship (6, 15).

## Final Considerations

In the terminal environment the effective communication is a valuable and powerful care weapon. There is a need to reinvent, research and, above all, reflect practices based on scientific rigor. The protocol to CBN suggested in this opinion paper aims to reduce the psychological distress of the health professional and foster discussion and improvement of assertive communication in the terminal environment due to the in the pandemic of COVID- 19. For, although the way in which palliative and end-of-life care is approached has changed dramatically with the pandemic, effective and honest communication must remain (3).

## Author Contributions

All authors prepared the review, developed the inclusion criteria, selected titles and abstracts, evaluated the quality of the articles included, and wrote the manuscript.

## Conflict of Interest

The authors declare that the research was conducted in the absence of any commercial or financial relationships that could be construed as a potential conflict of interest.
